# Partial lipodystrophy with severe insulin resistance and adult progeria Werner syndrome

**DOI:** 10.1186/1750-1172-8-106

**Published:** 2013-07-12

**Authors:** Bruno Donadille, Pascal D’Anella, Martine Auclair, Nancy Uhrhammer, Marc Sorel, Romulus Grigorescu, Sophie Ouzounian, Gilles Cambonie, Pierre Boulot, Pascal Laforêt, Bruno Carbonne, Sophie Christin-Maitre, Yves-Jean Bignon, Corinne Vigouroux

**Affiliations:** 1 Assistance Publique-Hôpitaux de Paris (AP-HP), Hôpital Saint-Antoine, Endocrinologie, Diabétologie et Endocrinologie de la Reproduction, F-75012, Paris, France; 2 Hôpital Henri Duffaut, Endocrinologie-Maladies Métaboliques, F-84902, Avignon, France; 3 INSERM UMR_S938, Centre de Recherche Saint-Antoine, F-75012, Paris, France; 4 UPMC Univ Paris 6, UMR_S938, F-75005, Paris, France; 5 ICAN Institute of Cardiometabolism and Nutrition, Groupe Hospitalier Universitaire La Pitié Salpêtrière, Paris, France; 6 Centre Jean Perrin, Laboratoire de diagnostic génétique et moléculaire, F-63000, Clermont-Ferrand, France; 7 Centre Hospitalier de Nemours, Centre d’Evaluation et de Traitement de la Douleur, and EA 4391, Faculté de médecine, Université Paris-Est Créteil, F-77796, Nemours, France; 8 Assistance Publique-Hôpitaux de Paris (AP-HP), Hôpital Armand-Trousseau, Génétique et Embryologie, F- 75012, Paris, France; 9 Hôpital A. de Villeneuve, Pédiatrie néonatale, F-34295, Montpellier, France; 10 Hôpital A. de Villeneuve, Gynéco-Obstétrique, F-34295, Montpellier, France; 11 Centre de Référence de pathologie neuromusculaire Paris-Est, Groupe Hospitalier Pitié-Salpêtrière, AP-HP, F-75013, Paris, France; 12 Assistance Publique-Hôpitaux de Paris (AP-HP), Hôpital Armand-Trousseau, Unité d'Obstétrique-Maternité, F-75012, Paris,France; 13 INSERM U933, F-75012, Paris, France; 14 AP-HP, Hôpital Tenon, Biochimie et Hormonologie, F-75020, Paris, France

**Keywords:** Lipodystrophy, Insulin resistance, *WRN* gene, Premature aging, Progeria, Pregnancy, Decreased ovarian reserve, Cervical insufficiency, Prelamin A, Lamin B1

## Abstract

**Background:**

Laminopathies, due to mutations in *LMNA*, encoding A type-lamins, can lead to premature ageing and/or lipodystrophic syndromes, showing that these diseases could have close physiopathological relationships. We show here that lipodystrophy and extreme insulin resistance can also reveal the adult progeria Werner syndrome linked to mutations in *WRN*, encoding a RecQ DNA helicase.

**Methods:**

We analysed the clinical and biological features of two women, aged 32 and 36, referred for partial lipodystrophic syndrome which led to the molecular diagnosis of Werner syndrome. Cultured skin fibroblasts from one patient were studied*.*

**Results:**

Two normal-weighted women presented with a partial lipodystrophic syndrome with hypertriglyceridemia and liver steatosis. One of them had also diabetes. Both patients showed a peculiar, striking lipodystrophic phenotype with subcutaneous lipoatrophy of the four limbs contrasting with truncal and abdominal fat accumulation. Their oral glucose tolerance tests showed extremely high levels of insulinemia, revealing major insulin resistance. Low serum levels of sex-hormone binding globulin and adiponectin suggested a post-receptor insulin signalling defect. Other clinical features included bilateral cataracts, greying hair and distal skin atrophy. We observed biallelic *WRN* null mutations in both women (p.Q748X homozygous, and compound heterozygous p.Q1257X/p.M1329fs). Their fertility was decreased, with preserved menstrual cycles and normal follicle-stimulating hormone levels ruling out premature ovarian failure. However undetectable anti-müllerian hormone and inhibin B indicated diminished follicular ovarian reserve. Insulin-resistance linked ovarian hyperandrogenism could also contribute to decreased fertility, and the two patients became pregnant after initiation of insulin-sensitizers (metformin). Both pregnancies were complicated by severe cervical incompetence, leading to the preterm birth of a healthy newborn in one case, but to a second trimester-abortion in the other. *WRN*-mutated fibroblasts showed oxidative stress, increased lamin B1 expression, nuclear dysmorphies and premature senescence.

**Conclusions:**

We show here for the first time that partial lipodystrophy with severe insulin resistance can reveal *WRN*-linked premature aging syndrome. Increased expression of lamin B1 with altered lamina architecture observed in *WRN*-mutated fibroblasts could contribute to premature cellular senescence. Primary alterations in DNA replication and/or repair should be considered as possible causes of lipodystrophic syndromes.

## Background

The studies of clinical and cellular consequences of *LMNA* mutations in humans have provided several indications of a close physiopathological relationship between premature aging and lipodystrophic syndromes. Indeed, human naturally-occuring *LMNA* mutations, among other phenotypes of laminopathies, have been shown to be responsible for premature ageing syndromes (Hutchinson-Gilford progeria, HGPS, and mandibuloacral dysplasia, MAD) [1-4], lipodystrophic syndromes (familial partial lipodystrophy of the Dunnigan type, FPLD2) [5,6], and mixed phenotypes [7-10]. At the cellular level, a highly similar disorganization of the nuclear lamina is observed in fibroblasts from patients with FPLD2, MAD and HGPS [3,11,12], including cellular replicative senescence and prelamin A accumulation [13-15]. Although the pathophysiological mechanisms leading to *LMNA*-linked premature aging are not fully understood, a major hypothesis is that alterations in maturation and farnesylation of lamin A induce genomic instability, abnormal epigenetic control of heterochromatin and DNA damage responses, and mesenchymal stem cell defects [16-18].

Werner syndrome (WS) is an autosomal recessive premature aging syndrome due to biallelic inactivating mutations in *WRN*, encoding a RecQ DNA helicase/exonuclease involved in DNA replication and repair [19]. Its prominent features, occurring after adolescence, associate “bird-like” facies, scleroderma-like skin changes with tight and atrophic skin, bilateral cataracts, short stature and premature greying of scalp hair [20]. An initial clinical presentation as a lipodystrophic syndrome has not been previously described.

In women with familial partial lipodystrophies, decreased fertility and obstetrical complications have been reported to be mainly linked to insulin resistance and metabolic disturbances, with an increased prevalence of polycystic ovary syndrome, gestational diabetes and eclampsia [21,22]. The mechanisms leading to decreased fertility in Werner syndrome have not been deciphered. Only rare cases of pregnancies have been reported in women with probable, but not genetically-confirmed Werner syndrome [23-26].

Here we report the cases of two women investigated for lipodystrophy and severe insulin resistance, which revealed Werner syndrome due to homozygous or compound heterozygous, non-sense or frameshift mutations in the *WRN* gene. The two patients became pregnant after initiation of metformin therapy. Both pregnancies were complicated by cervical incompetence, leading to a second-trimester abortion in one case and to a preterm delivery in the other patient. Cultured fibroblasts obtained from one patient showed cellular senescence, nuclear dysmorphies, and lamin staining abnormalities similar to those found in laminopathies, but did not overexpress immature prelamin A.

Our study points out that primary defects in DNA replication and/or repair should be considered as possible causes of lipodystrophic syndromes with extreme insulin resistance. In addition, we show that cell nuclear dysmorphies with alterations in lamin staining can be secondary to cellular senescence of different origin.

## Patients and methods

### Patients

Two women of 32 and 36 years old were referred to us for partial lipodystrophic syndrome. Clinical, biological, molecular, and cellular studies were performed after full written informed consent, according to the ethic committee of Hôpital Saint-Antoine, in line with the Helsinki Declaration.

### Phenotype and genotype characterization

Skinfolds thickness was measured using a Harpenden calliper. Routine serum measurements and 75g-oral glucose tolerance tests (OGTT) were performed after an overnight 12-h fast. Serum adiponectin and leptin levels were determined by ELISA (Quantikine, R&D Systems, Oxford, UK), on frozen-stored samples.

Genomic DNA from the two patients was extracted from peripheral blood leukocytes. The entire coding region and splice junctions of *LMNA, PPARG* and/or *WRN* were amplified and sequenced. DNA from patient 1’s placenta and from foetal liver and muscular tissues was extracted for direct sequencing of exon 19 of the *WRN* gene.

### Cellular studies

#### Cell cultures

Primary fibroblast cultures were established after skin biopsies in patient 1 and in two control women aged 20 and 33, without known disease, who underwent cosmetic surgery. Fibroblasts were grown in DMEM medium (Gibco® Cell Culture, Invitrogen Corporation, San Diego, CA, USA) containing 1 g/L glucose, 20 mM L-glutamine, 25 mM Hepes, 110 mg/ml sodium pyruvate, 10% foetal bovine serum (FBS, Gibco), 100 U/ml penicillin and 0.1 mg/ml streptomycin (Invitrogen Corporation) at 37°C in 5% CO2/95% air.

#### Western blot analysis

Western blot analyses were performed on whole cell extracts using antibodies directed against lamin-A/C (MAB-3211, Chemicon International Inc. Temecula, CA, USA), total prelamin-A (SC-6214, Santa Cruz Biotechnology, CA, USA), or uncleaved prelamin-A isoforms (i.e., containing the CSIM C-terminal aminoacids, farnesylated or not) (ANT0045, 1188–1, Diatheva, Fano, Italy) [27], lamin B1 (ab16048, Abcam, Cambridge, UK) and beta-actin (A5441; Sigma-Aldrich, Saint-Louis, MO, USA).

#### Cell morphology and immunofluorescence microscopy

Immunofluorescence studies were performed on fibroblasts grown on glass coverslips after fixation in methanol at −20°C, as previously described [15]. DAPI (4’,6’-di-amidine-2-phenylindole dihydrochloride) was used for nuclear staining. Antibodies directed against lamin-A/C (MAB-3211) and B type-lamins (a generous gift from JC. Courvalin and B. Buendia, Institut Jacques Monod, France [11,28]) were revealed by using secondary antibodies coupled to Texas Red or CyTM2 (Jackson Immuno Research Laboratories, West Grove, PA, USA). A total of 350 to 550 nuclei was examined and counted for each subject. Images were acquired and processed using Adobe Photoshop software after visualisation by conventional microscopy.

#### Reactive Oxygen Species (ROS) production

We used the CM-H2DCFDA derivatives (5- (and 6)-chloromethyl-2’,7’-dichlorodihydrofluorescein diacetate, acetyl ester, C6827, Molecular Probes) as cell-permeant indicators of ROS [15]. Cells were cultured in 96-well plates, then washed and incubated with CM-H2DCFDA (9 μM) or DAPI (0.01 μg/ml) in DMEM medium without FBS for 2 h at 37°C in the dark. Quantification, performed with a plate fluorescence reader (Spectrafluor Plus, Tecan-France, Trappes, France) at 520 nm (CM-H2DCFDA) or 465 nm (DAPI), was normalized to the DNA content.

#### Senescence-associated beta-galactosidase assay

Physiological lysosomal and senescence-associated beta-galactosidase activities were assessed by cellular X-gal blue staining at pH 4 and pH 6, respectively. Cells were fixed for 10 min at 22°C with 2% formaldehyde/0.2% glutaraldehyde, incubated overnight at 37°C in 1 mg/ml 5-bromo-4-chloro-3-indolyl-ß-D-galactoside (X-gal), 40 mM citric acid-sodium phosphate (pH 6 or 4), 5 mM potassium ferricyanide, 5 mM potassium ferrocyanide, 150 mM NaCl and 2 mM MgCl_2_, then lysed in H_2_O and sonicated. The blue X-gal cellular staining was quantified at 630 nm. The ratio of pH 6 to pH 4-staining specifically represented senescence-activated beta-galactosidase activity.

### Pathological and histological analyses

Macroscopic analyses of placenta and foetus were performed two days after abortion. Light microscopy was performed on 10% formol-fixed paraffin-embedded 5-mm tissue sections, stained with hematoxylin-eosin.

### Statistical analyses

All experiments were performed at least three times on triplicate samples. Quantitative results are expressed as mean ± SEM. Comparisons between *WRN*-mutated and control cells were made with Student’s t-test. *P* values of less than 0.05 were considered significant.

## Results

### Case reports

#### Patient 1

**Figure 1:**
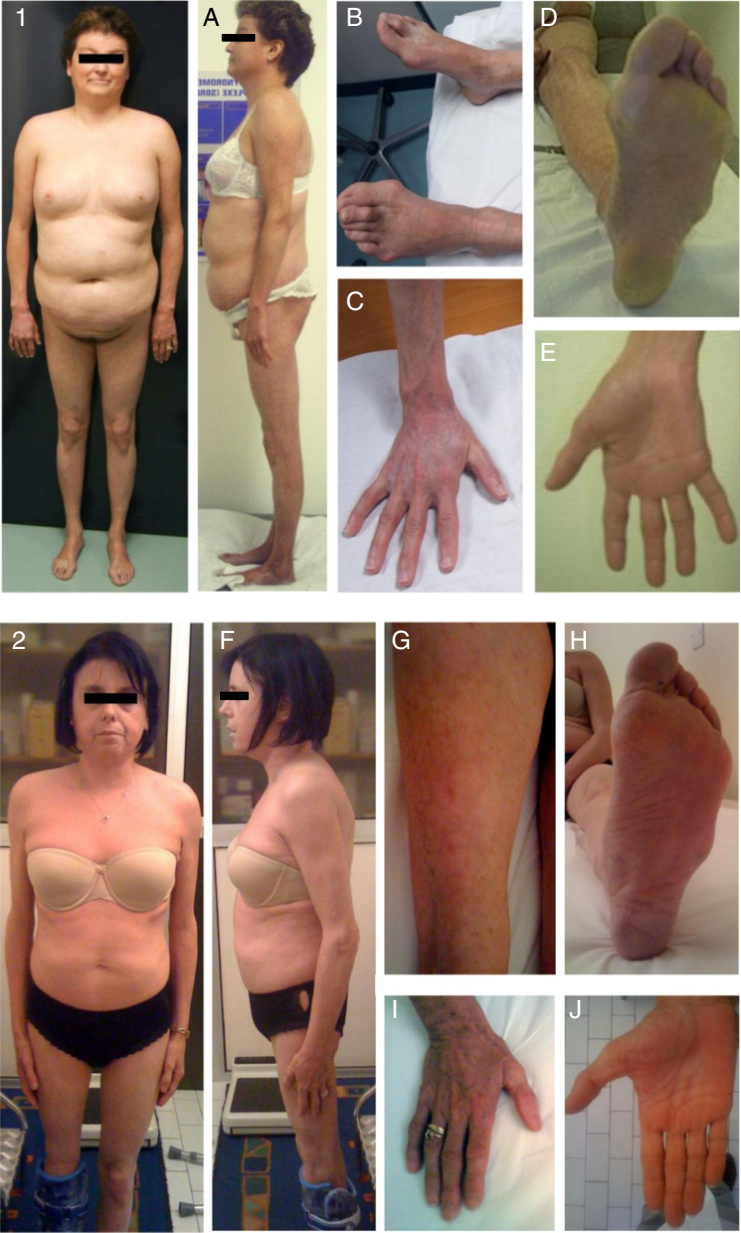
**Clinical features of patients.** Photographs of patient 1, aged 32 (1, **A, B, C, D, E**) and patient 2, aged 36 (2, **F, G, H, I, J**), showing partial lipodystrophy with subcutaneous fat accumulation of the trunk and abdomen with lipoatrophy of the limbs including palms and soles (1, **A, B, D, C, E**), livedo reticularis of the limbs (**B, D, G**), sclerodactylia (**C, E, I, J**), dry, tight and atrophic skin with hyperkeratosis of extremities (**B, C, D, E, H, I, J**).

A 32-year-old woman was referred to our Endocrinology Department for partial lipodystrophy. Her parents, without known consanguinity, originated from close villages of Madere Island, Portugal. Her medical history was uneventful until puberty, when she complained of myalgia during exercise. Pubertal development was normal with spontaneous menarche at age 11, followed by primary oligomenorrhea with menstrual cycles of 30 to 60 days. A first-trimester spontaneous miscarriage was reported at age 27. She had been treated a few months ago with amitryptiline then lamotrigine for painful peculiar peripheral neuropathy of the limbs (Sorel at al, manuscript in preparation). Physical examination showed lipoatrophy of the four limbs, including palms and soles, but sparing the face, and contrasting with truncal and abdominal subcutaneous fat accumulation (Figure 1). Peripheral lipoatrophy and central fat accumulation appeared progressively since puberty. Loss of muscle mass from the limbs was striking. Height was 158 cm, weight 60 kg (body mass index, BMI: 24 kg/m^2^), waist and hip perimeters 92 and 96 cm, respectively. Skin examination revealed livedo reticularis, dry, tight and atrophied skin, petechiae, purpuric lesions and hyperkeratosis, predominant in the extremities (Figure 1). No acanthosis nigricans or hirsutism were observed. Blood pressure was 120/70 mmHg. Cardiac examination was normal. Further inquiry revealed that hair graying appeared since age 25 and surgical extraction of bilateral cataracts at age 26. Among the five siblings of the patient, a brother died at 44 years old from myocardial infarction; he was reported with dysmorphic features, premature cataracts, insulin-treated diabetes since age 26, and severe atherosclerosis leading to foot amputation at age 38. A 42 yr-old sister died from sarcoma of the quadriceps femoris muscle.

**Figure 2:**
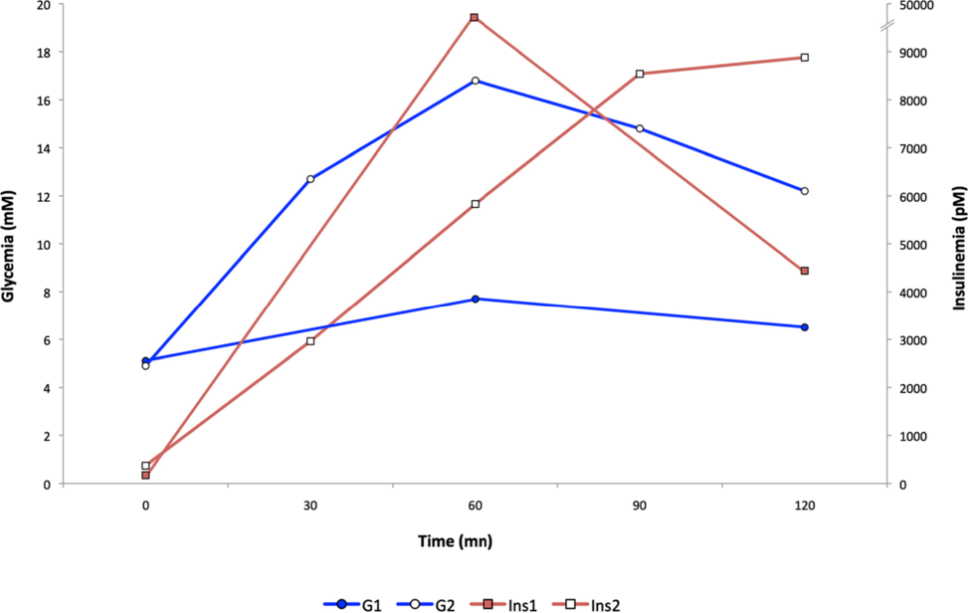
**Baseline oral glucose tolerance tests of patients, before metformin therapy.** G, glycemia (circles, blue), and Ins, insulinemia (squares, red) from patients 1 (filled symbols) and 2 (empty symbols) are depicted.

Biological investigations revealed severe insulin resistance with moderate fasting hyperinsulinemia (168 pmol/L, normal value < 100) reaching extremely high values after oral glucose tolerance test (OGTT) (maximal value 53800 pmol/L at T60 min, then 29750 pmol/L at T120 min, normal < 520), without altered glucose tolerance (Table 1 and Figure 2). Hypertriglyceridemia was associated with low HDL-cholesterol and slightly elevated alanine transaminase (ALT) and gamma-glutamyl transferase (GGT) levels. Sex-hormone binding globulin (SHBG) and adiponectin levels were low. Pituitary-ovarian functions and testosterone level were normal, but serum anti-müllerian hormone (AMH) and inhibin B were not detectable, suggesting decreased ovarian reserve (Table 1). Pelvic and transvaginal sonography showed a normal uterus but enlarged ovaries (right : 6.2 cm^2^, left : 6.9 cm^2^, normal volume < 5.5 cm^2^) with follicules of 10.4 to 15.8 mm.

**Table 1:** Biological features of patients

	**Patient 1**	**Patient 2**	**Normal values**
Fasting glycemia (mmol/L)	5.1	4.9	4-5.6
Fasting insulinemia (pmol/L)	168	371	13-100
2 h post-OGTT glycemia (mmol/L)	6.5	12.2	4–7.4
2 h post-OGTT insulinemia (pmol/L)	29750	8884	90–520
HbA1c (%)	6.8	6.1	< 5.5
Total cholesterol (mmol/L)	4.8	5.8	4-5.5
LDL-cholesterol (mmol/L)	3.6	3.5	1.2-4.1
HDL-cholesterol (mmol/L)	0.75	0.98	1-2
Triglycerides (mmol/L)	2.4	2.8	0.55-1.6
Alanine transaminase (ALT) (IU/L)	47	75	10-40
Aspartate transaminase (AST) (IU/L)	24	34	10-32
Gamma-glutamyl transferase (GGT) (IU/L)	60	113	8-54
Leptin (μg/L)	33	8.6	2.4-25
Adiponectin (mg/L)	0.8	1.9	3.9-12.9
Sex-hormone binding globulin (nmol/L)	14	16.1	40-80
Insulin-like growth factor 1 (IGF-1) (μg/L)	166	313	175-375 at age 30-40
Thyroid stimulating hormone (TSH) (mU/L)	4.8	3.8	0.8-4.8
Free T4 (μg/L)	9.4	8.6	7.5-21.1
Follicle stimulating hormone (FSH) (IU/L)	7.5	6.4	2-13
Luteinizing hormone (LH) (IU/L)	3.2	4.6	1.5-11
Estradiol (pmol/L)	215	480	80-370
Total testosterone (nmol/L)	0.96	1.7	0.3-1.85
Androstenedione (nmol/L)	4.9	13.5	2-11.4
Dehydroepiandrosterone sulfate (mmol/L)	2.4	1.8	0.9-11.6
Prolactin (μg/L)	6.6	5.4	3-27
Anti-Müllerian hormone (pmol/L)	<0.3	<0.3	3.6-62
Inhibin B (ng/L)	< 10	< 10	60-125

*OGTT* oral glucose tolerance test.

Hormone measurements were performed in early follicular phase.

Anti-Müllerian hormone and inhibin B levels were measured at age 33 for patient 1.

Electrocardiogram and dipyridamole-thallium myocardial scintigraphy were normal, whereas doppler ultrasonography evidenced a mildly increased carotid intima-media thickness (0.9 mm, normal < 0.6 mm). Other investigations revealed mild sensorineural hearing loss. A whole-body MRI did not reveal any neoplasm.

**Figure 3:**
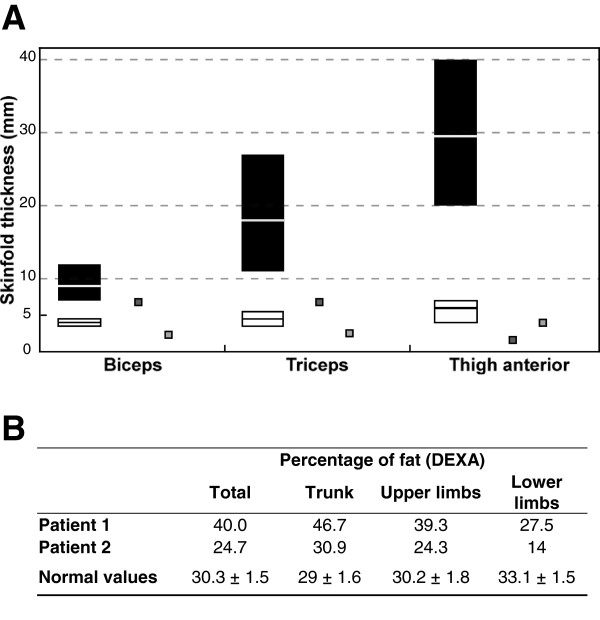
**Body fat distribution of patients: Skinfold thickness and whole-body dual-energy X-ray absorptiometry (DEXA).** (**A**) Skinfold thickness measured in patient 1 (dark grey squares) and patient 2 (light grey squares) as compared to that reported in typical FPLD2 women with *LMNA* p.R482 heterozygous substitutions (white rectangles) and control women (black rectangles) [29,30]. Median and range values are indicated. (**B**) Results of DEXA in patients 1 and 2, showing that body fat was predominantly distributed in the trunk area. Normal values (mean ± SD) are from [31].

**Figure 4:**
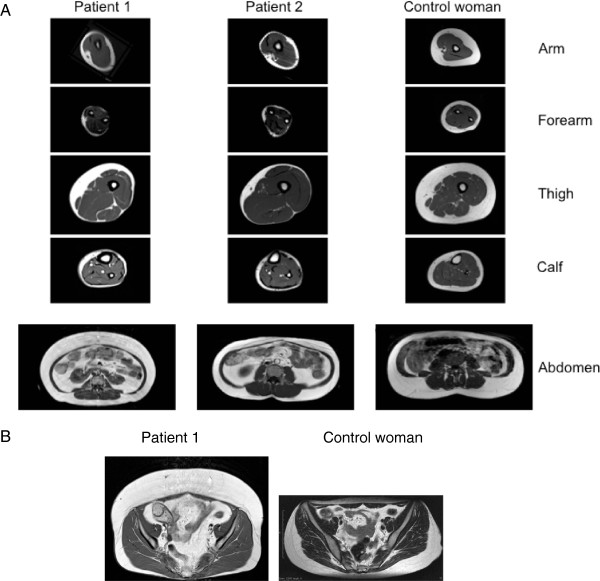
**Body fat distribution of patients: magnetic resonance imagery (MRI).** (**A**). T1-weighted MRI axial scans through the arm, forearm, thigh, calf and abdomen of patients 1 and 2, compared with a control woman of similar age (35 yr-old) and BMI (23 kg/m^2^). Partial lipoatrophy affecting the four limbs, contrasting with accumulation of intra-abdominal fat, was evidenced in both patients. Increased subcutaneous abdominal fat was also striking in patient 1. Bone marrow fat was well preserved. (**B**). T1-weighted MRI pelvic axial scan in patient 1 and in a control woman (BMI 21 kg/m^2^), showing lipoatrophy of gluteal fat depots with accumulation of anterior abdominal subcutaneous fat in patient 1.

Peripheral lipoatrophy was quantified by skinfold thickness measurements (Figure 3A). Whole-body dual-energy X-ray absorptiometry (DEXA) revealed increased body fat, predominantly distributed in the trunk area, in accordance with the clinical findings (Figure 3B). Bone mineralization was normal. MRI imaging showed that peripheral lipoatrophy affected the four limbs (Figure 4A). In the thighs, subcutaneous fat was asymmetrically distributed, with normal medial but reduced postero-lateral adipose tissue (Figure 4A). MRI also showed a severe loss of gluteal fat and an increase in both subcutaneous and visceral abdominal fat depots (Figure 4A and 4B). Imagery also revealed liver steatosis.

Sequencing of *LMNA* and *PPARG* did not reveal any alteration. *WRN* sequencing revealed homozygosity for a c.2242C>T transition in exon 19, predicting the synthesis of a p.Q748X WRN protein, truncated in its helicase domain, which was absent in 100 unrelated control subjects.

Appropriate diet and metformin therapy decreased insulin resistance, although insulinemia remained elevated (respectively 130 and 1600 pmol/l at OGTT T0 and T120 min after three months, with normal glycemia). Statin and fibrate treatments were poorly tolerated, due to muscular pain.

**Figure 5:**
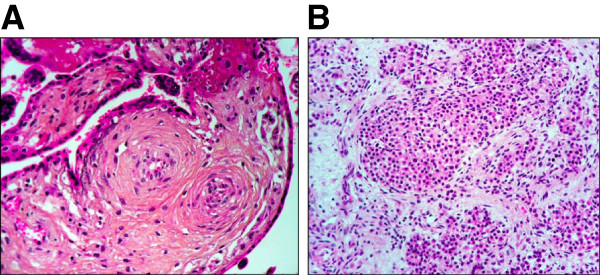
**Pathological features of placenta and foetus from patient 1 (hematoxylin-eosin staining).** (**A**) Placenta villosities examination. (**B**) Hyperplasia of Langerhans islet cells from the foetus’s pancreas.

One year later, after six years of infertility, the patient became spontaneously pregnant. She developed gestational diabetes, treated with diet and insulin. At 10 weeks of pregnancy, a short cervix (18 mm) was noted and a cervical cerclage was performed. Obstetrical ultrasound scan was normal at 16 weeks, but premature rupture of membranes, followed by spontaneous abortion occurred at 20 weeks of gestation. Pathological examination of the placenta showed signs of chorioamnionitis and funiculitis, which did not allow precise evaluation of possible previous vascular alterations (Figure 5A). The female foetus weighted 294 g (normal for gestational age), and showed a craniofacial dysmorphy associating retrognatism, receding forehead and flat nasal bridge, that could be linked to prolonged oligohydramnios due to premature rupture of membranes. X-ray analysis of the whole skeleton was normal. Foetal histopathological examination only revealed hyperplasia of pancreatic Langerhans islet cells (Figure 5B). Heterozygosity for the p.Q748X *WRN* mutation was observed in placental and foetal DNA.

#### Patient 2

A 36-year-old woman was referred for partial lipodystrophy. She was born from non-consanguineous healthy parents of Sicilian and French origin. Her two siblings, a 34-yr old sister and a 38-yr old brother, were also reported to be healthy. Her medical history was uneventful until age 30, when she underwent surgical removal of a chondrosarcoma of the right distal femur (with total knee prosthetic replacement). At this time a 2 g/l fasting glycemia was observed, in the absence of antibodies directed against glutamic acid decarboxylase 65 and tyrosine phosphatase (anti-GAD and anti-IA2); BMI was 20.3 kg/m^2^. Hypertriglyceridemia reached 4 g/l, and liver steatosis was diagnosed on ultrasonographic appearance and elevated ALT and GGT in the absence of any other cause. Metabolic alterations were controlled using diet, fibrates and α-glucosidase inhibitors. Regarding gynecological history, puberty was normal, with spontaneous menarche at age 11, and regular menstrual cycles of 28 days. A first-trimester spontaneous miscarriage was reported at age 34. Since then, she did not conceive and consulted for 2 year infertility. At examination, she presented a partial lipodystrophy phenotype, with lipoatrophy and loss of muscle mass of the four limbs, palms and soles, which progressively appeared since age 20. Lipoatrophy spared the face, which contrasted with truncal and abdominal subcutaneous fat accumulation (Figure 1 and Figure 3A). Height was 144 cm, weight 39 kg (BMI: 18.8 kg/m^2^), waist and hip perimeters 76 cm. A high-pitched voice was noted. Skin examination revealed livedo reticularis on the limbs, with dry, tight, atrophied skin and hyperkeratosis, predominant in the extremities (Figure 1). No acanthosis nigricans or hirsutism were observed. Blood pressure was 110/60 mmHg. Cardiac examination was normal but doppler monitoring revealed diffuse atherosclerosis. Further inquiry revealed that hair graying appeared since age 12, and bilateral cataracts were diagnosed at age 34.

OGTT revealed severe hyperinsulinemia (fasting and T120 min values, 371 and 8884 pmol/L, respectively) with diabetes (glycemia was normal at fast but reached 12.2 mmol/L at T120 min OGTT) (Table 1 and Figure 2). Hypertriglyceridemia was associated with slightly low HDL-cholesterol and elevated ALT and GGT. Leptin was normal for BMI. SHBG and adiponectin levels were low. FSH and testosterone levels were normal, but AMH and inhibin B very low (Table 1).

Peripheral lipoatrophy was assessed by skinfold thickness measurements (Figure 3A) and by MRI imaging. As for patient 1, MRI showed asymmetrical distribution of subcutaneous fat in the thighs and increased intra-abdominal fat stores (Figure 4). DEXA confirmed that fat was predominant in the trunk and reduced in the lower limbs (Figure 3B). Bone mineral density was low (−2 and −2.8 SD at the vertebral and the femoral levels, respectively).

*WRN* sequencing revealed previously undescribed compound heterozygous mutations, with a c.3769C>T transition in exon 32 predicting a p.Q1257X truncation, and a c.3986delT frameshift deletion in exon 34 predicting the synthesis of a 1333 amino acid p.M1329fs WRN truncated protein, both mutated proteins lacking their nuclear localization signal. These mutations were absent in 100 unrelated control subjects.

Metformin was added to the treatment of the patient, and she became pregnant two months later. Insulin therapy was needed from the second month of pregnancy. A prophylactic cervical cerclage was performed at 15 weeks of gestation, but premature rupture of membranes with preterm labor occurred at 21 weeks. Treatment allowed the maintenance of pregnancy until 31 weeks, when a caesarean section was performed for chorioamnionitis. The patient gave birth to a healthy female child of 2310 g (normal for gestational age) with normal clinical examination except for the presence of a receding forehead without radiological signs of craniodysostosis. X-ray analysis of the whole skeleton, and cardiac and abdominal echography were normal. Placental histological examination confirmed the diagnosis of chorioamnionitis but did not reveal other specific alterations.

### Cell studies

Primary fibroblast cultures from patient 1 were studied in comparison to control cells and/or to cells from a patient with FPLD2 due to the heterozygous p.R482W *LMNA* mutation [15], at the same passage.

#### Fibroblasts from patient 1 showed nuclear shape abnormalities without altered expression and localization of type A lamins

**Figure 6:**
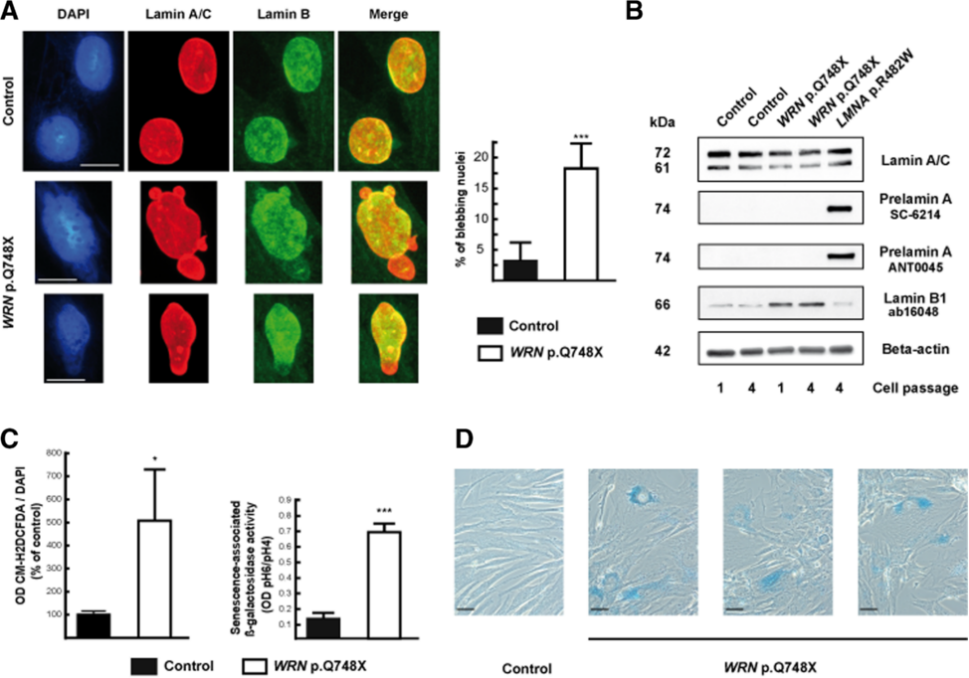
***WRN*-mutated fibroblasts showed nuclear shape abnormalities, oxidative stress and premature senescence, without altered expression and localization of type A lamins.** Cultured skin fibroblasts from patient 1 were compared to control cells. (**A**) Nuclear staining of fibroblasts was performed with diamidino-2phenylindole hydrochloride (DAPI) (DNA, blue), and with antibodies directed against lamin A/C (red), and lamin B (green). Representative cells, studied at passages 4 to 8, are shown. Lamin A/C staining was heterogeneous and lamin B staining reduced or absent in nuclear blebs or poles of dysmorphic *WRN*-mutated cells, but not in control cells. Scale bars represent 10 μm. Quantification of cells with dysmorphic nuclei was performed after examination of 200 to 250 cells for each subject. Results are expressed as mean ± SEM. ***, p < 0.001. (**B**) Fibroblast lysates were submitted to Western blot, using antibodies which specifically detect lamin A/C (MAB3211), lamin B1 (ab16048), prelamin A (SC-6214), and uncleaved prelamin A (having retained its CSIM C-terminal tail) (ANT0045). Beta-actin was used as a loading control. Representative results from control, *WRN*-mutated, and p.R482W *LMNA*-mutated cells, used as positive controls [15], are shown as indicated. (**C**) ROS production was assessed by oxidation of CM-H_2_DCFDA derivatives, normalized to the DNA content, and senescence-associated ß-galactosidase cell activity by the ratio of X-gal staining at pH6 and pH4, in *WRN*-mutated and control fibroblasts studied at the same passage (2 to 5). Results from four experiments performed in quadriplate are shown, as mean ± SEM. *, p < 0.05, ***, p < 0.001. (**D**) Representative micrographs of cells stained with X-gal at pH 6 are shown. Control cells were studied at passage 1 to 4, and *WRN*-mutated cells at passage 1. Note the senescence-associated blue staining, flattened and enlarged morphology of *WRN*-mutated cells. Scale bars represent 40 μm.

Nuclear shape abnormalities, with lobulations and blebs, were more prevalent in patient 1’s than in controls’ cells, studied at the same early passage (mean ± SEM : 18.4 ± 4.1 vs 3.4 ± 3.2%, respectively, p<0.001). In addition, nuclear blebs showed altered lamin A/C staining and were frequently devoid of lamin B, with a weak DNA staining suggesting chromatin decondensation, as shown by immunocytochemistry (Figure 6A). Protein expression of type A-lamins was not different, but lamin B1 levels were increased in fibroblasts from patient 1, as compared to control or *LMNA* p.R482W-mutated cells. Prelamin A, which is known to be accumulated in p.R482W *LMNA*-mutated cells [15], was not detectable in control and patient 1’s cells (Figure 6B).

#### Fibroblasts from patient 1 showed increased oxidative stress and premature senescence

Oxidative stress, assessed by cellular ROS production, was increased in fibroblasts from patient 1 as compared to controls, at the same passage (Figure 6C). The *WRN-*mutated fibroblasts also prematurely acquired a senescence-associated flattened and enlarged morphology in culture, and have a significantly increased senescence-associated ß-galactosidase activity, as compared to control cells of the same passage (Figure 6D).

## Discussion

In the present study, we add further evidence for a pathophysiological link between cellular senescence and lipodystrophy, by showing for the first time that partial lipodystrophy syndrome with extreme insulin resistance can be the initial referring presentation of the adult progeria Werner syndrome, due to a primary defect in the WRN enzyme, involved in DNA replication and repair.

Lipodystrophic syndromes are heterogeneous diseases of genetic or acquired origin, characterized by generalized or partial lipoatrophy associated with insulin resistance. Recent advances in molecular genetics have shown that primary defects in fat differentiation and/or adipose lipid droplet formation or maintenance are the main causes of genetic lipodystrophies (for review, see [32-34]). However, the hypothesis that lipodystrophy could also be secondary to primary mesenchymal cellular senescence was raised by the studies of laminopathies, which collectively name a group of diseases due to alterations in the ubiquitous nuclear intermediate filaments A type-lamins, encoded by the *LMNA* gene. Indeed, mutations in *LMNA* can lead to Dunnigan-type familial partial lipodystrophy (FPLD2) [5,6], but also to accelerated ageing syndromes [1-4], and to mixed overlapping phenotypes with both lipoatrophy, metabolic complications and progeroid signs [7-10]. In addition, cellular studies have shown that, although lamin A alterations could impair adipogenesis through mislocalisation of the key adipogenic transcription factor SREBP1c [14,35-37], premature cellular senescence probably also participates to the pathophysiology of *LMNA*-linked lipodystrophy [15].

Werner syndrome, also called adult progeria (OMIM 277700), is one of several progeroid syndrome due to defective DNA helicases (for review, see [20]). This segmental aging syndrome, affecting several organ systems, is due to recessive null mutations in the WRN protein, which exhibits exonuclease, ATPase and helicase activities. Cellular senescence associated with Werner syndrome has been linked to DNA replication and repair defects [19]. The clinical diagnostic criteria have been defined by the International Registry of Werner Syndrome (http://www.wernersyndrome.org/registry/diagnostic.html) [20], and have been recently revised on the basis of the results of a Japanese nationwide epidemiological survey [38].

Both patients described here were referred for partial lipodystrophy, which is not listed as a classical sign of the disease. Several lipodystrophic features were different to those usually observed in other types of partial lipoatrophic syndromes, as FPLD2 or 3 due to *LMNA* or *PPARG* mutations, respectively. Indeed, in both patients, peripheral lipoatrophy was associated with loss of limb muscles, which contrasted with the muscle hypertrophy associated with FPLD2 and 3. In addition, marked central adiposity was striking, and imagery revealed an asymmetrical distribution of subcutaneous fat in the thighs. Both patients also exhibited several cardinal signs of Werner disease, *i.e.* bilateral cataracts, tight and atrophied skin with hyperkeratosis, and premature greying of scalp hair. In addition, patient 2 had short stature, and two siblings of patient 1 were probably affected, although molecular analysis was not possible. Further signs, listed as reminiscent of Werner disease, were also present: atherosclerosis and altered fertility in both cases, and high-pitched voice, diabetes, osteoporosis and mesenchymal neoplasm in patient 2. In both patients, we identified truncating null mutations affecting both alleles of *WRN* gene with loss of the nuclear localization signal. The homozygous p.Q748X *WRN* mutation of patient 1 was previously found in a Caucasian man diagnosed with Werner syndrome, but his clinical features were not reported [20], whereas patient 2 was affected by new *WRN* mutations. No evident genotype-phenotype correlations have been reported in Werner syndrome [20,39], although proximal truncation of WRN protein could lead to severe phenotypes [40]. Further studies are needed to eventually link the lipodystrophic clinical presentation to specific *WRN* mutations. However, our report shows that Werner syndrome is an important differential diagnosis in patients initially presenting with partial lipodystrophy as a prominent feature, leading to a specific follow-up, in particular regarding cancer risk, gynecology, and genetic counseling.

These two women presented with severe insulin resistance, with insulinemia being dramatically increased during OGTT without hypoglycemia. Low SHBG and adiponectin levels suggest a post-receptor insulin signalling defect, as observed in other lipodystrophic syndromes, where ectopic fat deposition, particularly in muscle and liver, is thought to play a major role in insulin resistance [41]. In accordance, our patients had also liver steatosis and hypertriglyceridemia. Their peripheral skinfolds confirmed a severe subcutaneous lipoatrophy of the limbs, whereas percentage of fat measured by DEXA in limbs was increased or only slightly decreased, suggesting that the limb muscles could be infiltrated with lipids. Insulin resistance has been previously reported in patients with Werner syndrome, with hypoadiponectinemia and increased intra-abdominal visceral fat in some patients [42,43], but insulin values did not reach such dramatically elevated levels [43,44]. Therefore, our report suggests that the presence of a marked lipodystrophy can contribute to the severity of insulin resistance in Werner syndrome.

Our report also points out the specific gynecological and obstetrical complications of Werner syndrome. Hypogonadism is a classic sign of Werner syndrome, but its precise origin has not been investigated [20,38]. To our knowledge, only three pregnant women with clinically-suspected, but not genetically-confirmed Werner syndrome, have been previously reported [23-26]. None of them carried their pregnancies to full-term, with spontaneous abortions at 10, 16 and 23 weeks [23], or preterm delivery at 25 to 34 weeks of gestation, due to cervical insufficiency in three cases [23,24] or to caesarean section for maternal life-threatening ischemic heart disease in one case [25,26]. Preeclampsia occurred during two pregnancies, in the absence of diabetes [23], and placental vascular alterations were observed in one case [24]. Our two patients had experienced early spontaneous abortions and had reduced fertility. They both became pregnant during the follow-up, but severe cervical incompetence, which thus appears to be a frequent obstetrical complication in this disease, led to a preterm birth in one case, and to a second term-abortion in the other. No specific abnormality was evidenced in the foetus and the preterm newborn. Pancreatic islet hyperplasia observed at foetal autopsy was linked to gestational diabetes. The placenta showed signs of chorioamnionitis and funiculitis, which were secondary to premature rupture of membranes.

In these women, reduced fertility was probably related to a premature decrease in the pool of primordial ovarian follicles (diminished ovarian reserve). Indeed, although their menstrual cycles were not modified, and their FSH levels were still within normal range, indicating that they did not had premature ovarian failure, their AMH and inhibin B serum levels were very low [45]. Biological ovarian hyperandrogenism, observed in patient 2, and polycystic ovaries in patient 1, favored by extreme insulin resistance, could also have contributed to decreased fertility. In accordance, both pregnancies were obtained after reducing hyperinsulinemia with metformin treatment. Therefore, both insulin resistance and premature ovarian ageing could lead to decreased fertility in Werner syndrome.

Lipodystrophic and progeroid laminopathies are characterized by cellular senescence and nuclear dysmorphies, with nuclear blebs showing abnormal nuclear staining of A and B-type lamins [3,11,12,15,46]. Several studies have suggested that accumulation of farnesylated forms of prelamin A could underlie these abnormalities (for review, see [47]). Our present results show that fibroblasts with WRN null mutations present nuclear deformations similar to those observed in laminopathies, as described in one previous study [48]. We also showed that oxidative stress and cellular senescence were enhanced in *WRN*-mutated cells. However, prelamin A was not accumulated in *WRN*-mutated cells, suggesting that primary DNA repair defects and *LMNA* alterations act by different mechanisms to induce premature ageing. In addition, although lamin B staining was decreased in nuclear blebs, the overall lamin B1 protein expression was increased in *WRN*-mutated cells, which was not previously described in laminopathies. The role of B type-lamins in cellular senescence is complex [49]. Indeed, replicative or oncogene-induced senescence has been linked to underexpression of lamin B1 [50], but oxidative stress-induced senescence to overexpression of lamin B1 [51]. An interesting hypothesis is that cellular stresses which alter the normal ratio of lamin B1 to functional lamin A, lead to deleterious changes in nuclear lamina, then senescence [49]. In accordance, and in line with our results, cells from patients with ataxia-telangiectasia, another genetic disease due to DNA damage signalling defects, display a state of endogenous oxidative stress which induces lamin B1 overexpression, nuclear shape alterations and senescence through MAP kinase activation [51]. It is possible that MAP kinase activation, which was previously observed in Werner syndrome [52], could also contribute to lamin B1-linked cellular senescence. Whether *WRN*-linked lipodystrophy could be secondary to primary mesenchymal cellular senescence, as it was suggested in laminopathies, needs to be further investigated.

## Conclusion

This report points out that partial lipodystrophic syndrome with severe insulin resistance can reveal Werner syndrome due to *WRN* mutations. Therefore, the search for clinical and biological arguments in favor of this diagnosis is mandatory in patients with partial lipodystrophies. In addition to some clinical features that distinguish lipodystrophic syndromes due to *WRN* as compared to *LMNA* mutations (as early cataracts and mesenchymal neoplasms), peculiar gynecological and obstetrical risk factors, *i.e.* diminished ovarian reserve and cervical incompetence, also characterize women with Werner syndrome. From a cellular point of view, this study shows that *WRN* mutations induce alterations in type B lamins expression and localization, with nuclear dysmorphies, that could participate to cellular senescence.

## Abbreviations

HGPS: Hutchinson-Gilford progeria; MAD: Mandibuloacral dysplasia; FPLD2: Familial partial lipodystrophy of the Dunnigan type; WS: Werner syndrome; OGTT: Oral glucose tolerance test; DAPI: 4’,6’-di-amidine-2-phenylindole dihydrochloride; ROS: Reactive oxygen species; CM-H2DCFDA derivatives: 5- (and 6)-chloromethyl-2’,7’-dichlorodihydrofluorescein diacetate, acetyl ester; X-gal: 5-bromo-4-chloro-3-indolyl-ß-D-galactoside; BMI: Body mass index; ALT: Alanine transaminase; GGT: Gamma-glutamyl transferase; SHBG: Sex-hormone binding globulin; AMH: Anti-müllerian hormone; DEXA: Whole-body dual-energy X-ray absorptiometry.

## Competing interests

The authors declare that they have no competing interests.

## Authors’ contributions

BD cared for one of the patients, analyzed the data, contributed to the discussion and drafted the manuscript. PD’A, MS, SO, GC, PB, PL, BC and SC-M cared for one of the patients, contributed to the discussion and reviewed the manuscript. MA carried out the cellular studies and contributed to the discussion, NU performed molecular analyses, contributed to the discussion and reviewed the manuscript. Y-JB performed molecular analyses, cared of one of the patient, contributed to the discussion and reviewed the manuscript. RG performed pathological analyses, contributed to the discussion and reviewed the manuscript. CV cared of the patients, analyzed the data, and wrote the manuscript. All authors read and approved the final manuscript.
